# Characterization of the Complete Uric Acid Degradation Pathway in the Fungal Pathogen *Cryptococcus neoformans*

**DOI:** 10.1371/journal.pone.0064292

**Published:** 2013-05-07

**Authors:** I. Russel Lee, Liting Yang, Gaseene Sebetso, Rebecca Allen, Thi H. N. Doan, Ross Blundell, Edmund Y. L. Lui, Carl A. Morrow, James A. Fraser

**Affiliations:** 1 Australian Infectious Diseases Research Centre, University of Queensland, Brisbane, Queensland, Australia; 2 School of Chemistry and Molecular Biosciences, University of Queensland, Brisbane, Queensland, Australia; University of Minnesota, United States of America

## Abstract

Degradation of purines to uric acid is generally conserved among organisms, however, the end product of uric acid degradation varies from species to species depending on the presence of active catabolic enzymes. In humans, most higher primates and birds, the urate oxidase gene is non-functional and hence uric acid is not further broken down. Uric acid in human blood plasma serves as an antioxidant and an immune enhancer; conversely, excessive amounts cause the common affliction gout. In contrast, uric acid is completely degraded to ammonia in most fungi. Currently, relatively little is known about uric acid catabolism in the fungal pathogen *Cryptococcus neoformans* even though this yeast is commonly isolated from uric acid-rich pigeon guano. In addition, uric acid utilization enhances the production of the cryptococcal virulence factors capsule and urease, and may potentially modulate the host immune response during infection. Based on these important observations, we employed both *Agrobacterium-*mediated insertional mutagenesis and bioinformatics to predict all the uric acid catabolic enzyme-encoding genes in the H99 genome. The candidate *C. neoformans* uric acid catabolic genes identified were named: *URO1* (urate oxidase), *URO2* (HIU hydrolase), *URO3* (OHCU decarboxylase), *DAL1* (allantoinase), *DAL2,3,3* (allantoicase-ureidoglycolate hydrolase fusion protein), and *URE1* (urease). All six ORFs were then deleted via homologous recombination; assaying of the deletion mutants' ability to assimilate uric acid and its pathway intermediates as the sole nitrogen source validated their enzymatic functions. While Uro1, Uro2, Uro3, Dal1 and Dal2,3,3 were demonstrated to be dispensable for virulence, the significance of using a modified animal model system of cryptococcosis for improved mimicking of human pathogenicity is discussed.

## Introduction

**Figure 1:**
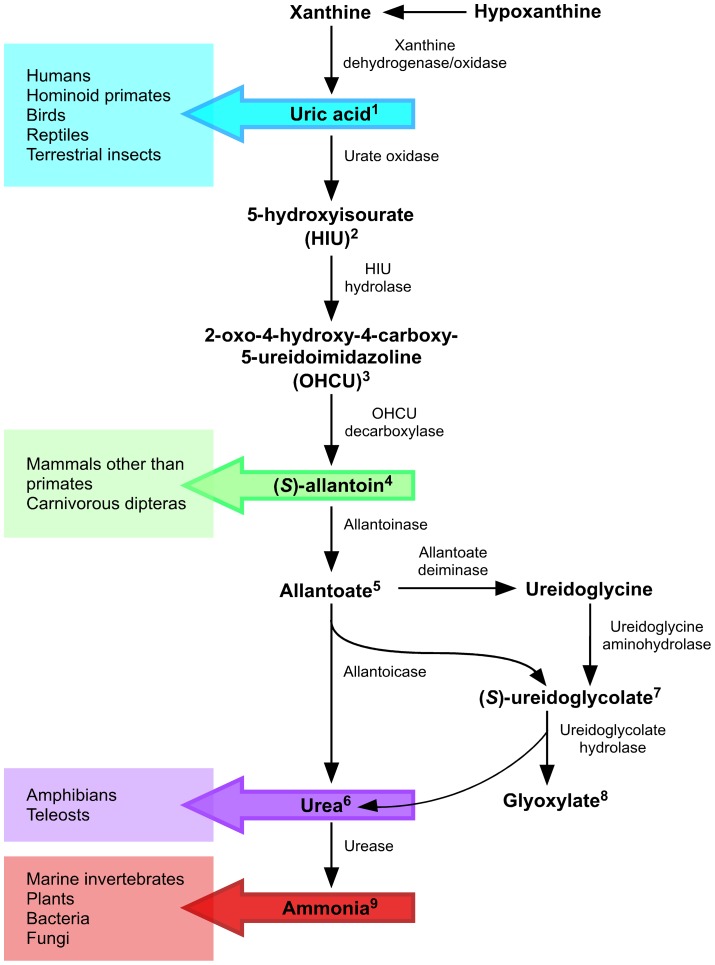
The end product of purine metabolism varies from species to species. While degradation of purines to uric acid is generally conserved among organisms, the end product of uric acid catabolism varies among taxa often due to the loss of functional catabolic enzymes in the pathway.

Purine catabolism is generally well conserved throughout organisms ranging from animals and fungi to plants and bacteria. While the degradation of purines to uric acid^1^ is common to all kingdoms of life, the resulting uric acid can either be excreted or further degraded in the peroxisomes by active uric acid catabolic enzymes (Figure 1). The various nitrogenous end products in different organisms are a result of the successive loss-of-function mutations in urate oxidase (also known as uricase), allantoinase and allantoicase during evolution. For instance, humans and apes share the inability to breakdown uric acid with Dalmatian dogs, birds, reptiles and insects [1], [2]. As uric acid is a potent antioxidant in the human blood plasma and protects cells from damage by scavenging reactive oxygen species, it has been proposed that the loss of urate oxidase may have been selected for due to it promoting longevity [3]. Despite these protective advantages, uric acid is poorly soluble in the serum and can precipitate to cause ailments such as gout or urate kidney stones [4].

Until recently, the conversion of uric acid to allantoin had long been thought to involve a single step catalyzed by urate oxidase [2]. However, production of allantoin is in fact preceded by two additional distinct unstable intermediates: 5-hydroxyisourate (HIU)^2^ and 2-oxo-4-hydroxy-4-carboxy-5-ureidoimidazoline (OHCU)^3^. HIU can spontaneously decompose to OHCU and subsequently racemic allantoin at a slow, non-enzymatic rate *in vitro* [5], [6], [7]. However, only dextrorotatory allantoin, (*S*)-allantoin^4^, is usually found in living cells [2]. The two additional biological enzymes, HIU hydrolase and OHCU decarboxylase, are responsible for the quicker *in vivo* hydrolysis of HIU to OHCU and the decarboxylation of OHCU to (*S*)-allantoin, respectively [8], [9]. Most mammals, except higher primates, have lost both allantoinase and allantoicase and hence excrete allantoin as the end product.

Some organisms however, do have the ability to further catabolize (*S*)-allantoin to exploit its stored nitrogen, carbon and energy. Allantoinase catalyzes the hydrolysis of (*S*)-allantoin to allantoate^5^, allantoicase catalyzes the degradation of allantoate to urea^6^ and ureidoglycolate^7^, while ureidoglycolate hydrolase degrades ureidoglycolate to glyoxylate^8^ and another molecule of urea. In most amphibians and fishes, purine degradation proceeds to urea and glyoxylate as the end products [10].

Interestingly, in plants and in some bacteria (enterobacteria and *Bacillus* spp.), an alternative allantoate metabolic route also exists: apart from the urea-releasing route, the other results in the production of ammonia and carbon dioxide [2], [11], [12], [13], [14], [15], [16], [17], [18], [19]. Allantoate deiminase is responsible for the catalysis of allantoate for the ammonia-releasing route, while the urea-releasing route necessitates subsequent urea hydrolytic activity by urease or urea amidolyase for full catabolism to ammonia^9^. Most plants, bacteria and fungi (an exception occurs in the model yeast *Saccharomyces cerevisiae* where the pathway does not begin until the step of allantoin degradation) generally possess all the necessary nitrogen catabolic enzymes to completely degrade uric acid to ammonia. Hence, ammonium is the most readily assimilated nitrogen source that is preferentially utilized by most bacteria and fungi in order to conserve energy. In the presence of ammonium, the expression of permease and catabolic enzyme-encoding genes required to assimilate non-preferred nitrogen sources is usually repressed, a regulatory mechanism known as nitrogen catabolite/metabolite repression [20], [21], [22].

Understanding the uric acid degradation pathway in *Cryptococcus neoformans*, a fungal pathogen that commonly causes fatal meningitis in the immunocompromised population, is clinically relevant [23]. The *C. neoformans* primary ecological niche is purine-rich pigeon droppings, and the majority of the nitrogen present is in the form of uric acid, with the rest consisting primarily of xanthine, urea and creatinine [24]. In addition, the antiphagocytic polysaccharide capsule is a well-known virulence factor that is induced in the presence of uric acid as the sole nitrogen source [25], [26]. Assimilation of uric acid necessitates numerous catabolic enzymes including another well-established virulence factor, urease, which plays a role in central nervous system invasion during host infection [27], [28], [29]. Incidentally, both uric acid and urea are common metabolites found at high concentration in the human blood. In fact, necrotic mammalian cells release uric acid as a danger signal to activate both innate and adaptive immune effectors including neutrophils, cytotoxic T-cell and antibody immunity [30].

The association between uric acid availability in the niches occupied by *C. neoformans* and how its utilization influences virulence factor expression and potential modulation of host immune response, prompted us to characterize the uric acid catabolic enzymes. Every year, up to a million immunocompromised individuals are infected with *C. neoformans*, two-thirds of whom will die [31]. Due to the poor outcomes associated with the treatment of cryptococcosis, the cost of treatment and the toxicity of the pharmaceutical agents currently available, it is imperative that we identify new therapeutic agents to help combat this disease. Rational drug design was pioneered in the purine metabolic pathway over half a century ago, and has been the paradigm for targeted drug discovery since the development of allopurinol and thioguanine for the treatment of gout and leukaemia, respectively [32]. Humans have successively lost the uric acid enzymes during species evolution; should the *C. neoformans* uric acid degradation pathway regulate pathogenesis and provide a viable drug target, there exist over 50 years worth of literature describing innumerable compounds designed to modulate the activity of these enzymes. In this study, we genetically and phenotypically characterized the functions of all six of the *C. neoformans* uric acid catabolic enzyme-encoding genes in relation to nitrogen source utilization and virulence-associated mechanisms.

## Materials and Methods

### Strains and media

All fungal strains used in this study are listed in Table S1, and were grown in YPD (1% yeast extract, 2% Bacto-peptone, 2% glucose) or YNB (0.45% yeast nitrogen base w/o amino acids and ammonium sulfate, 2% glucose, 10 mM nitrogen source) unless specified otherwise. *C. neoformans* biolistic transformants were selected on YPD medium supplemented with 200 µg/mL G418 (Sigma) or 100 µg/mL nourseothricin (Werner BioAgents). The 12% (wt/vol) pigeon guano medium was prepared as described previously, with the exclusion of the filtration step to ensure that insoluble nitrogen sources such as uric acid would not be removed [33]. V8 (5% V8 juice, 3 mM KH_2_PO_4_, 0.1% *myo*-Inositol, 4% Bacto-agar) and Murashige-Skoog (MS) mating media (*Phyto*Technology Laboratories) at pH 5.0, and L-3,4-dihydroxyphenylalanine (L-DOPA) medium at pH 5.6, were prepared as described previously [34], [35], [36], [37]. *Escherichia coli* Mach-1 cells served as the host strain for transformation and propagation of all plasmids using lysogeny broth supplemented with either 100 µg/mL ampicillin (Sigma) or 50 µg/mL kanamycin (Sigma) [38]. *Caenorhabditis elegans* strain N2 was maintained at 15°C and propagated on its normal laboratory food source *E. coli* OP50 cells [39], [40], [41]. Nematode growth medium (NGM) was prepared as described previously [39].

### Bioinformatic analyses

*C. neoformans* genes were identified using annotation from the H99 genome sequence from the Broad Institute (http://www.broadinstitute.org/annotation/genome/cryptococcus_neoformans/MultiHome.html). Gene annotations from the Broad are designated by their nomenclature “CNAG_#####.#”. Sequence analyses were performed using BLAST and MacVector 9.5 (MacVector Inc, Cary NC) [42]. Sequence alignments were created using ClustalW v1.4 within MacVector [43]. Sequence traces generated at the Australian Genome Research Facility (Brisbane, Queensland) were analyzed using Sequencher 4.7 (Gene Codes Corporation, Ann Arbor MI). Putative peroxisomal localization signals were examined using Target Signal Predictor Tool (http://www.peroxisomedb.org/Target_signal.php).

### Construction and screening of library for *C. neoformans* uric acid catabolism defective mutants

An *Agrobacterium-*mediated mutagenesis library of *C. neoformans* H99 was created as described previously [44]. The majority of transformants harbored a single copy of randomly integrated T-DNA and was mitotically stable. To identify genes involved in uric acid catabolism, replica spotting of the insertional library consisting of 12,000 clones was carried out on YNB supplemented with uric acid or ammonium (10 mM each nitrogen source). Mutants that grew on ammonium but failed to grow on uric acid-containing medium were selected for further analysis. T-DNA insertion site was mapped using GenomeWalker according to the manufacturer's instructions (Clontech). BLAST analysis of these sequences was performed to reveal which loci were affected.

### Construction and complementation of *C. neoformans* mutant strains

All primers and plasmids used in this study are listed in Table S2 and S3, respectively. Gene deletion mutants were created using overlap PCR and biolistic transformation as described previously [45]. For example, to construct the *uro1Δ* mutant strain in the H99 background, the 1,389 bp *URO1* (CNAG_04307.2) coding sequence was replaced with the neomycin phosphotransferase II-encoding selectable marker *NEO* using a construct created by overlap PCR combining a ∼1 kb fragment upstream the *URO1* start codon, the *NEO* marker and a ∼1 kb fragment downstream the *URO1* stop codon. Strain H99 genomic DNA and plasmid pJAF1 were used as PCR templates [46]. The construct was transformed into *C. neoformans* cells via particle bombardment and transformants were selected on YPD plates supplemented with G418. Deletion of *URO1* was confirmed by diagnostic PCR and Southern blot [47]. To complement the *uro1Δ* mutant, the *URO1* gene including ∼1 kb promoter and terminator was amplified from genomic DNA using high fidelity PCR, cloned into pCR2.1-TOPO (Invitrogen) to give pIRL5, and sequenced. The *URO1* fragment of pIRL5 was then subcloned into pCH233, creating the complementation construct pIRL13. pIRL13 was subsequently linearized and biolistically transformed into the *uro1Δ* mutant. Stable transformants were selected on YPD supplemented with nourseothricin and complemented strains containing a single copy of the wild-type *URO1* gene were identified by Southern blot.

### Mating assays

Mating assays were conducted as described previously [33]. Briefly, strains were suspended in PBS either alone or mixed in equal proportions with a strain of the opposite mating-type (*MAT*). Droplets (5 µL) were then spotted onto V8, MS or pigeon guano medium [33], [35], [37]. Plates were incubated at room temperature in the dark for 1 week and assessed by light microscopy for the formation of filaments and basidia.

### Capsule assays

Strains were inoculated into RPMI supplemented with 10% fetal calf serum (Gibco) and incubated at 37°C for 2 days. To visualize capsule, cells were stained with India ink (Becton Dickinson) and observed under a ZEISS Axioplan 2 epifluorescent/light microscope. Pictures were taken with an Axiocam greyscale digital camera using the AxioVision AC imaging software.

### Growth and melanization assays

Starter *C. neoformans* cultures were prepared by growth in YPD at 30°C overnight with shaking, diluted to OD_595nm_  = 0.05 in water, then further diluted tenfold in series. Each diluted cell suspension was then spotted onto YPD, YNB supplemented with the specified nitrogen source or L-DOPA agar. Results were imaged after 2–3 days incubation at 30°C (nitrogen utilization assays), 30 and 37°C (melanization assays), or 37–39°C (high temperature growth assays).

### *C. elegans* killing assays

Starter cultures of *C. neoformans* strains were prepared by growth in YPD at 30°C overnight with shaking. Overnight cultures (10 µL) were spread onto 35 mm brain-heart infusion (BHI) (Becton Dickinson) agar plates, and incubated at 25°C overnight. Approximately 50 young adult *C. elegans* worms were then transferred from a lawn of *E. coli* OP50 on NGM to BHI medium-grown *C. neoformans* [48]. Plates were incubated at 25°C and worms examined for viability at 24-hr intervals using a dissecting microscope, with worms that did not respond to a touch with a platinum wire pick considered dead. Each experimental condition was performed in triplicate. Survival was plotted against time, and *P* values were calculated by plotting a Kaplan-Meier survival curve and performing a log-rank (Mantel-Cox) test using Graphpad Prism Version 5.0c. *P* values of <0.05 were considered statistically significant.

### Murine inhalation model of cryptococcosis

For murine virulence assays, *C. neoformans* were used to infect 6-week old female BALB/c mice by nasal inhalation [27]. For every tested strain, ten mice were each inoculated with a 50 µL drop containing 5×10^5^ cells. Mice were weighed before infection and daily thereafter; animals were sacrificed using CO_2_ inhalation once their body weight had decreased to 80% of the pre-infection weight. Survival was plotted against time, and *P* values were calculated by plotting a Kaplan-Meier survival curve and performing a log-rank (Mantel-Cox) test using Graphpad Prism Version 5.0c. *P* values of <0.05 were considered statistically significant.

### Ethics statement

This study was carried out in strict accordance with the recommendations in the Australian Code of Practice for the Care and Use of Animals for Scientific Purposes by the National Health and Medical Research Council. The protocol was approved by the Molecular Biosciences Animal Ethics Committee of The University of Queensland (AEC approval number: SCMB/008/11/UQ/NHMRC). Infection was performed under methoxyflurane anaesthesia, and all efforts were made to minimize suffering through adherence to the Guidelines to Promote the Wellbeing of Animals Used for Scientific Purposes as put forward by the National Health and Medical Research Council.

## Results

### *Agrobacterium-*mediated insertional mutagenesis reveals several enzymes required for uric acid assimilation in *C. neoformans*

Insertional mutagenesis is a powerful tool for identifying novel genes and their functions, and the frequency of random integration is very high if *Agrobacterium tumefaciens-*mediated transformation is used. A T-DNA insertional library of *C. neoformans* strain H99 was therefore created using this transkingdom DNA delivery approach in an attempt to locate loci involved in uric acid utilization. We replica spotted 12,000 purified individual transformants onto minimal YNB medium supplemented with uric acid or ammonium (control) as the sole nitrogen source, and mapped the T-DNA insertion site of 8 disrupted mutants that grew on ammonium but exhibited impaired growth on uric acid (Table S4). Our sequence analysis revealed four independent ORFs that play a role in uric acid catabolism. Three of these genes were predicted to encode well-established catabolic enzymes known in other systems to be necessary for uric acid degradation (urate oxidase, allantoicase and urease), while one was predicted to encode the less familiar HIU hydrolase only recently proposed to be part of this pathway.

### Bioinformatic analyses predict the complete uric acid degradation pathway of *C. neoformans*

Using the existing knowledge of purine catabolism in model ascomycetes, bioinformatic analyses were performed to complete the entire predicted uric acid degradation pathway of the basidiomycete *C. neoformans.* Protein sequences of characterized *Aspergillus nidulans* or *S. cerevisiae* uric acid catabolic enzymes were queried against the H99 genome using reciprocal BLASTp searches. The candidate *C. neoformans* uric acid catabolic enzyme-encoding genes identified were named: *URO1* (CNAG_04307.2), *URO2* (CNAG_06694.2), *URO3* (CNAG_00639.2), *DAL1* (CNAG_00934.2), *DAL2,3,3* (CNAG_01108.2), and *URE1* (CNAG_05540.2). Our previous work has shown that the expression of at least two of these putative catabolic genes, *URO1* and *DAL1*, is inducible by the presence of uric acid (pathway-specific induction) and repressible by the presence of ammonium (nitrogen metabolite repression) [25].

The *URO1* ORF is predicted to encode the urate oxidase protein of 305 amino acid residues, and comparative analysis of Uro1 with the *A. nidulans* homolog UaZ revealed 42% identity and 54% similarity (Figure S1). Importantly, the conserved N-terminus of eukaryotic uricases that is critical for enzymatic activity is highly similar between Uro1 and UaZ. The *URO2* ORF is predicted to encode the HIU hydrolase protein of 122 amino acids, and an alignment with the *A. nidulans* homolog UaX revealed 32% identity and 47% similarity (Figure S2). Both Uro2 and UaX contain the conserved C-terminal tetrapeptide YRGS, a signature of the thyroid hormone transporter transthyretin which probably originated from a duplication of an ancestral HIU hydrolase gene [49], [50]. Additionally, both Uro2 and UaX show complete conservation of all residues involved in the binding of the substrate in characterized HIU hydrolases [50], [51], [52]. The *URO3* ORF is predicted to encode the OHCU decarboxylase protein of 204 residues, and an alignment with the *A. nidulans* homolog UaW revealed 26% identity and 43% similarity (Figure S3). Notably, peroxisomal targeting signals were not detected in Uro1, Uro2 and Uro3 using Target Signal Predictor, providing no indication on their subcellular localization. Thus, Uro1, Uro2 and Uro3 may possibly rely on another unidentified peroxisomal targeting sequence, as previously demonstrated in the *A. nidulans* glyoxylate cycle enzyme isocitrate lyase (AcuD) that was shown to be peroxisomal without any obvious targeting signal peptide [53].

*URO1, URO2* and *URO3* homologs are absent in *S. cerevisiae*, however, the genes encoding proteins required to complete the remaining steps of this pathway are arrayed in the largest metabolic cluster known in yeast [54], [55]. Allantoin degradation is best studied in *S. cerevisiae* and genes in this *DAL* cluster include *DAL1* (allantoinase), *DAL2* (allantoicase), *DAL3* (ureidoglycolate hydrolase), *DAL4* (allantoin permease), *DCG1* (allantoin racemase) and *DAL7* (malate synthase that reduces the toxicity of allantoin catabolism via disposal of excess glyoxylate). Analysis of the H99 genome revealed that homologs of all these *DAL* genes are present but are not clustered in a single chromosome.

The *C. neoformans DAL1* ORF is predicted to encode the allantoinase protein of 478 amino acids, and an alignment with the *S. cerevisiae* homolog Dal1 revealed 43% identity and 59% similarity (Figure S4). Interestingly, homology to *S. cerevisiae DAL2* and *DAL3* in *C. neoformans* is represented by a single ORF encoding a fusion allantoicase-ureidoglycolate hydrolase protein of 813 residues. In fact, closer inspection revealed two tandemly arrayed Dal3 products (Figure S5A). The existence of this plausible fusion event, presumably encoding a bifunctional enzyme, was confirmed by the acquisition of cDNA that spanned the entire ORF, disproving the possibility of gene clustering. This phenomenon is only present in the phylum *Basidiomycota* and is not observed in the *Ascomycota* or *Zygomycota,* indicating that the fusion event occurred 500 million – 1 billion years ago [56]. An alignment of *C. neoformans* Dal2 with the *S. cerevisiae* homolog Dal2 revealed 36% identity and 52% similarity (Figure S5B). An alignment of *C. neoformans* Dal3a (first paralog) with the *S. cerevisiae* homolog Dal3 revealed 24% identity and 38% similarity, while *C. neoformans* Dal3b (second paralog) showed 23% identity and 40% similarity (Figure S5C).

**Figure 2:**
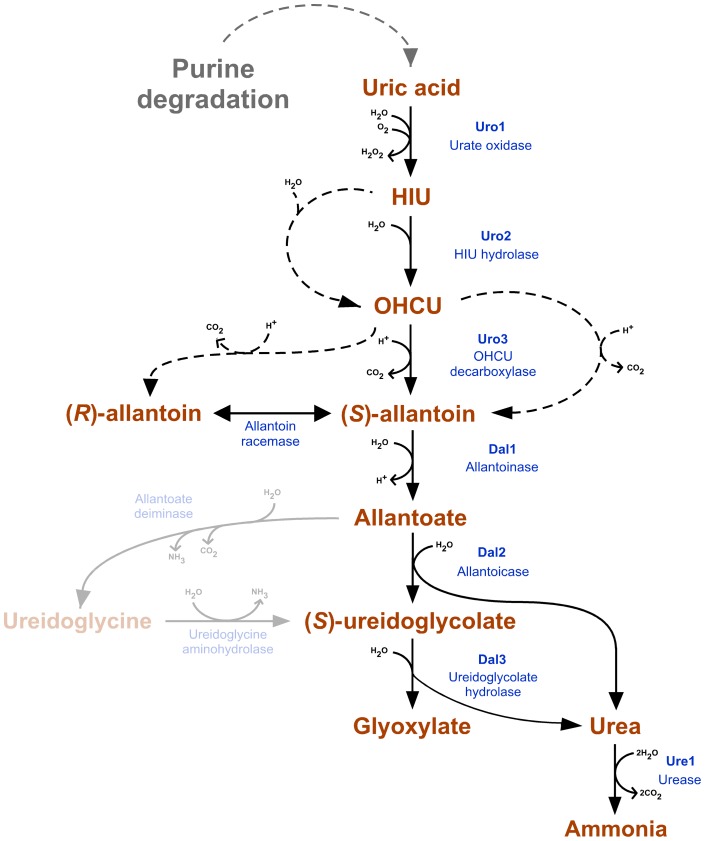
Predicted pathway of uric acid degradation in *C. neoformans*. While the metabolic pathway of purine degradation is present in bacteria, fungi, plants and animals, there is hidden complexity between species. In addition to species in which only part of the pathway is present, these variations include at least three examples of convergent evolution (conversion of xanthine to uric acid, allantoin to allantoate, and urea to ammonia), offshoots of the pathway that are only present in some species (allantoin racemase, ureidoglycine production and hydrolysis) as well as points at which spontaneous conversion occur without the need for enzyme activity (dashed arrows). Steps predicted to be absent from *C. neoformans* are opaque.

Finally, the *URE1* ORF that encodes the nickel-containing urease of 833 amino acids is the only enzyme of the *C. neoformans* uric acid degradation pathway that has been characterized, due to its precedence in fungi and bacteria as an important pathogenic factor [27], [28], [29]. An alignment of *C. neoformans* Ure1 with the *A. nidulans* homolog UreB revealed 60% identity and 75% similarity (Figure S6). In *S. cerevisiae,* urea utilization occurs via the *DUR1,2-*encoded urea amidolyase which is absent in *C. neoformans* [57]. Altogether, based on random insertional mutagenic screen and exhaustive bioinformatics, the predicted uric acid degradation pathway of *C. neoformans* is deciphered (Figure 2).

### Verification of the catabolic functions of the putative *C. neoformans* uric acid enzymes using reverse genetics

**Figure 3:**
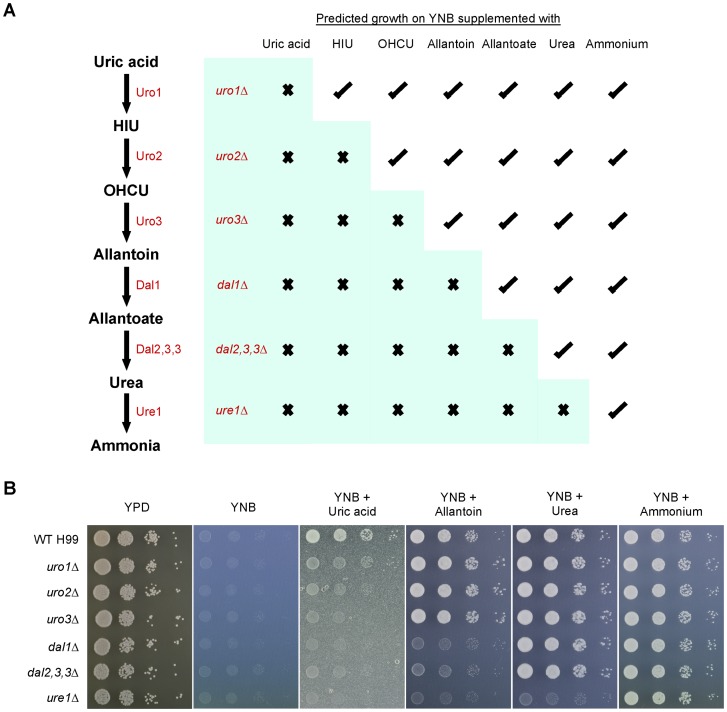
Nitrogen assimilation phenotypes of the *C. neoformans* uric acid catabolic deletion mutants. (A) Based on the enzymatic activity of homologs in other systems, inactivation of each respective catabolic enzyme (in red) is predicted to disrupt the ability of *C. neoformans* to utilize one or more pathway intermediates as the sole nitrogen source. (B) Ten-fold spot dilution assays on YNB supplemented with 10 mM uric acid or its derivatives revealed that the *uro1Δ, uro2Δ, uro3Δ, dal1Δ, dal2,3,3Δ* and *ure1Δ* mutants showed a correlation with the anticipated nitrogen utilization abilities.

To verify the functions of *URO1, URO2, URO3, DAL1, DAL2,3,3* and *URE1*, gene deletion mutants were created via homologous recombination in strain H99. All the deletion strains were viable and their growth appeared indistinguishable from wild-type on rich YPD medium. Growth of the set of deletion mutants was then tested on YNB medium supplemented with uric acid and its pathway intermediates as the sole nitrogen source (Figure 3). However, growth on HIU, OHCU and allantoate were not tested either due to the compound's chemical instability (HIU and OHCU) or discontinued production by biotechnological companies (allantoate).

As expected, loss of each respective gene resulted in inability of the mutant to retain the enzymatic activity required to degrade its related purine derivative. The *uro1Δ, uro2Δ* and *uro3Δ* mutants exhibited growth defects on uric acid, but displayed wild-type growth on allantoin, urea and ammonium. While the *uro2* and *uro3* mutations resulted in impaired growth on uric acid after a two-day incubation period, growth however catches up on further incubation, consistent with the fact that spontaneous conversion of HIU to OHCU and subsequently to allantoin can still occur at a slow, non-enzymatic rate in the absence of HIU hydrolase or OHCU decarboxylase (Figure S7). The *dal1Δ* and *dal2,3,3Δ* mutants exhibited growth defects on uric acid and allantoin, but displayed wild-type growth on urea and ammonium. Lastly, the *ure1Δ* mutant exhibited growth defects on uric acid, allantoin and urea, but displayed wild-type growth on ammonium. Complementation of all the uric acid catabolic deletion mutants restored the wild-type nitrogen utilization phenotypes, verifying the enzymatic functions of Uro1, Uro2, Uro3, Dal1, Dal2,3,3 and Ure1 (Figure S8).

### None of the *C. neoformans* uric acid catabolic enzymes play a role in the initiation of mating

**Figure 4:**
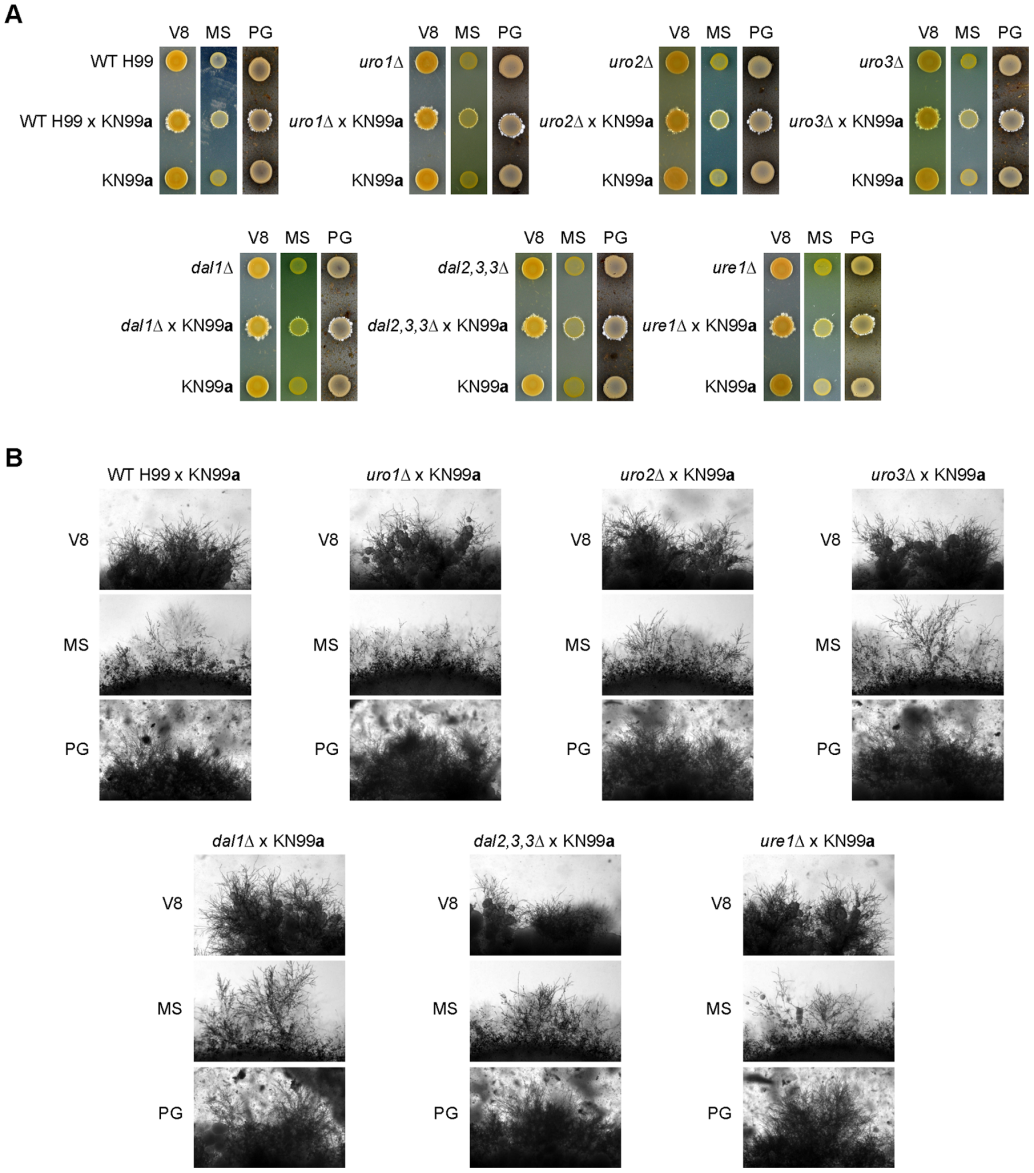
Uro1, Uro2, Uro3, Dal1, Dal2,3,3 and Ure1 are not required for *C. neoformans* initiation of mating. Filamentation assays on V8, MS and pigeon guano (PG) medium showed that filament formation appeared indistinguishable between the crosses of H99 × KN99**a** and each of the uric acid mutants × KN99**a**. (A) Periphery of each individual colony. (B) Under higher magnification (×400).

Uric acid-rich pigeon guano is a common source for infectious propagules of *C. neoformans* and is postulated to play a key part in transmission from the environment to a human host [23], [24]. Quite recently, *C. neoformans* was shown to undergo robust sexual reproduction on medium containing sterilized pigeon guano extracts in the laboratory [33]. To address whether the uric acid catabolic enzymes play a role in this sexual development, we unilaterally crossed the wild-type and mutant strains of H99α background with the opposite mating-type, KN99**a**, on V8, MS and pigeon guano medium (Figure 4). Production of filaments and basidia at the edges of the mating spots were indistinguishable between the crosses of H99 × KN99**a** and each of the uric acid mutants × KN99**a** on the various mating medium, suggesting that the uric acid enzymes are not likely to play a role in the initiation of morphological differentiation of the *C. neoformans* life cycle. Further bilateral mating crosses may be performed to support this conclusion.

### None of the *C. neoformans* uric acid catabolic enzymes contribute to the production of capsule or melanin, or the ability to grow at high temperature

**Figure 5:**
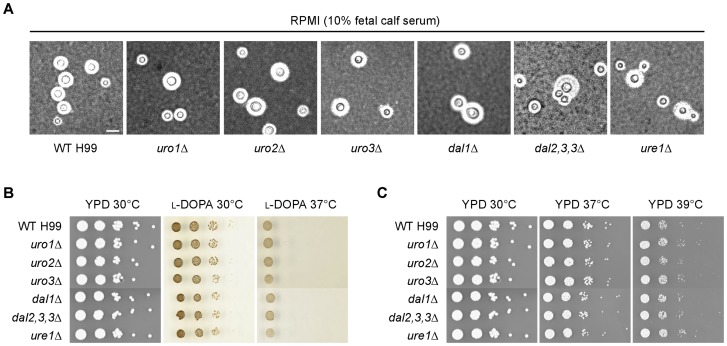
Uro1, Uro2, Uro3, Dal1, Dal2,3,3 and Ure1 are not required for expression of the three major virulence attributes. (A) India ink cell staining under light microscopy revealed that the uric acid catabolic deletion mutants produced characteristic halos around its cells representing enlarged capsule that were similar to wild-type when cultured under serum-induced growth conditions. Scale bar, 10 µm. (B) Ten-fold spot dilution assays on L-DOPA medium at both 30 and 37°C showed that the catabolic deletion mutants melanized to the same extent as wild-type. **C.** Ten-fold spot dilution assays on YPD medium at human body temperature (37 and 39°C) demonstrated that the deletion mutants exhibited wild-type growth.

As the influence of uric acid on certain cryptococcal virulence factor expression has been established, we investigated whether any of the uric acid catabolic enzymes affect the production of the antiphagocytic capsule or antioxidant melanin, and the ability to grow at human body temperature (Figure 5) [25], [26]. The *uro1Δ, uro2Δ, uro3Δ, dal1Δ, dal2,3,3Δ* and *ure1Δ* mutants all displayed these three classical virulence traits that were indistinguishable from wild-type, suggesting that the uric acid enzymes are dispensable for virulence factor expression when alternative nitrogen sources are available to compensate for the lack of uric acid utilization. This result reiterates the complex regulation of virulence factor production that is influenced by multiple physiological conditions.

### HIU hydrolase activity is required for *C. neoformans* killing of nematode worms

**Figure 6:**
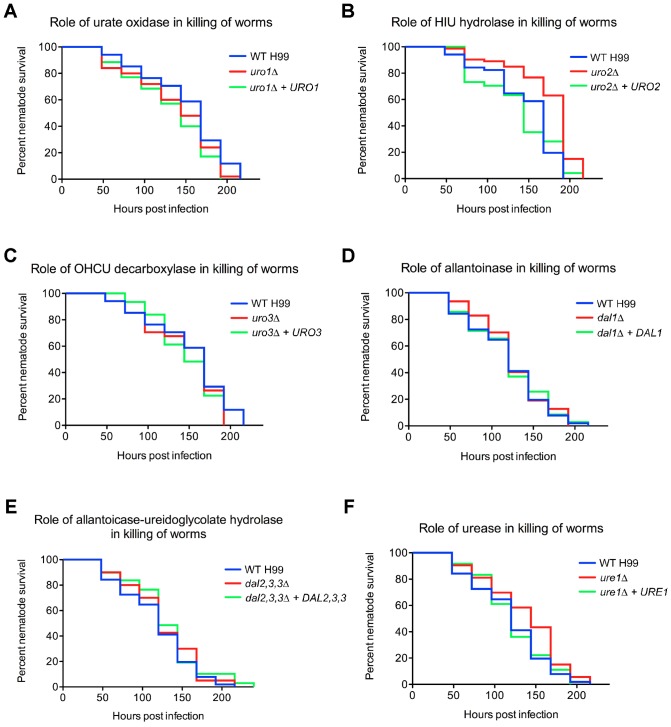
HIU hydrolase plays a subtle role in *C. neoformans-*mediated killing of *C. elegans.* ∼50 nematode worms were transferred to a lawn of (A) wild-type H99, *uro1Δ, uro1Δ + URO1,* (B) *uro2Δ, uro2Δ + URO2,* (C) *uro3Δ, uro3Δ + URO3,* (D) *dal1Δ, dal1Δ + DAL1,* (E) *dal2,3,3Δ, dal2,3,3Δ + DAL2,3,3,* (F) *ure1Δ* or *ure1Δ + URE1,* as the sole food source on BHI medium and survival was monitored at 24-hr intervals. There was no observable difference in *C. elegans* killing by the *uro1Δ, uro1Δ + URO1, uro2Δ + URO2, uro3Δ, uro3Δ + URO3, dal1Δ, dal1Δ + DAL1, dal2,3,3Δ, dal2,3,3Δ + DAL2,3,3, ure1Δ* and *ure1Δ + URE1* strains compared to wild-type. In contrast, the *uro2Δ* strain killed *C. elegans* slightly slower than wild-type. All experiments were repeated three times with similar results.

There have been many instances in the *Cryptococcus* literature whereby various mutant strains show no reduction in traits normally associated with virulence *in vitro,* yet significantly impact pathogenicity *in vivo* [58], [59]. *C. neoformans* presumably interacts with *C. elegans* in the environment and killing of *C. elegans* by *C. neoformans* has previously been validated as a model for studying fungal pathogenesis [48]. We performed *C. elegans* virulence assays using standard BHI medium to evaluate if any of the uric acid catabolic enzymes contribute to *C. neoformans* pathogenesis (Figure 6). Killing of *C. elegans* by the *uro1Δ, uro3Δ, dal1Δ, dal2,3,3Δ* and *ure1Δ* strains was not significantly different to that observed for the wild-type strain, indicating that urate oxidase, OHCU decarboxylase, allantoinase allantoicase-ureidoglycolate hydrolase and urease are not required for *C. neoformans-*mediated killing of invertebrate worms. However, the *uro2Δ* mutant killed *C. elegans* slightly less efficient than the wild-type strain (WT vs *uro2Δ, P*<0.0001), implicating HIU hydrolase in *C. neoformans* virulence. The median survival of the worms infected with wild-type in comparison to *uro2Δ* is 7 and 8 days, respectively.

### None of the *C. neoformans* uric acid catabolic enzymes, with the exception of urease, is required for pathogenesis during infection of a mammalian host

**Figure 7:**
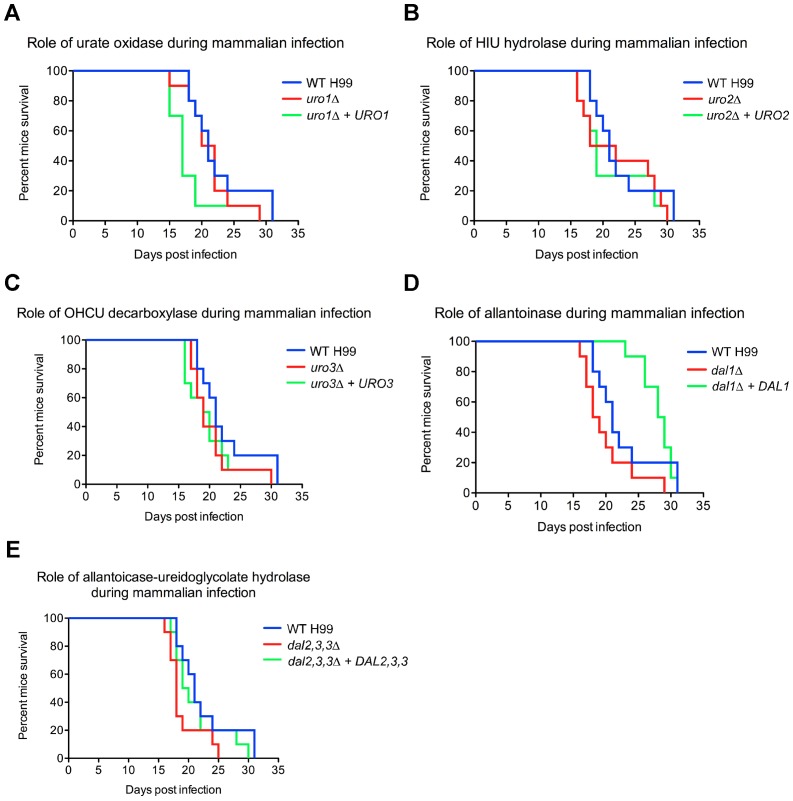
Uro1, Uro2, Uro3, Dal1 and Dal2,3,3 are not required for infection of a murine host. 10 mice were each intranasally infected with either 5×10^5^ cells of (A) wild-type H99, *uro1Δ, uro1Δ +URO1,* (B) *uro2Δ, uro2Δ +URO2,* (C) *uro3Δ, uro3Δ + URO3,* (D) *dal1Δ, dal1Δ +DAL1,* (E) *dal2,3,3Δ* or *dal2,3,3Δ + DAL2,3,3,* and survival was monitored daily. Mice infected with the *uro1Δ, uro1Δ +URO1, uro2Δ, uro2Δ +URO2, uro3Δ, uro3Δ + URO3, dal1Δ, dal2,3,3Δ* and *dal2,3,3Δ + DAL2,3,3* strains progressed to morbidity as quickly as mice infected with the wild-type strain. Unexpectedly, the complemented *dal1Δ +DAL1* strain appeared to be significantly less virulent than wild-type; this conundrum is likely caused by integration of *DAL1* into a non-native, virulence-associated (possibly high temperature-associated) locus.

We next performed a murine inhalation model of cryptococcosis, widely regarded as the gold standard test for virulence assays of *C. neoformans,* to examine the roles of urate oxidase, HIU hydrolase, OHCU decarboxylase, allantoinase and allantoicase-ureidoglycolate hydrolase during infection of a mammalian host (Figure 7). The virulence-associated function of urease has already been thoroughly characterized in several different animal models of *C. neoformans* infection and hence it is omitted from this study [27], [28], [29]. Surprisingly, mice intranasally infected with the *uro1Δ, uro2Δ, uro3Δ, dal1Δ* and *dal2,3,3Δ* mutant strains all succumbed to infection at approximately the same rate as mice infected with the wild-type strain (killing occurred between 18 and 31 days post infection, median survival of mice  = 21 days). In order to provide parsimonious explanations to the conflicting *uro2Δ* results in the *C. elegans* killing assays and the experimental murine cryptococcosis, investigating the exact mechanism by which *C. neoformans* kills *C. elegans* might be beneficial; while accumulation of the fungus within the gastrointestinal tract of the worm is known to be associated with killing, the precise nature of the cause of death is unclear [48].

## Discussion

Nitrogen scavenging through the uric acid degradation pathway is complex and it involves multiple enzymatic steps. While this pathway is conserved in bacteria, fungi, plants and animals, it nevertheless displays wondrous variation not only between kingdoms of life, but also from species to species. In the soil bacterium *Bacillus subtilis,* eight genes [*pucL* and *pucM* (uricases), *pucJ* and *pucK* (uric acid transporters), *pucH* (allantoinase), *pucI* (allantoin permease), *pucF* (allantoate amidohydrolase), *pucR* (transcriptional activator of *puc* gene expression)] whose products act in concert to drive uric acid catabolism, are all located in a cluster with the exception of *pucI* [14]. In the model hemiascomycete *S. cerevisiae,* genomic rearrangements have led to the assembly of six adjacent genes of the *DAL* cluster that enables preferential utilization of allantoin as a nitrogen source over assimilation of uric acid that requires the oxygen-consuming urate oxidase [55]. Incidentally, baker's yeast can grow vigorously in anaerobic conditions.

Such gene clustering for purine metabolism is not observed in filamentous ascomycetes such as *A. nidulans,* where the entire uric acid degradation pathway has recently been dissected [8]. The discovery of a different form of gene clustering (the gene-fusion event involving *DAL2,3,3*) in *C. neoformans* introduces a new paradigm to the fungal uric acid degradation pathway. It is not uncommon for certain protein families in a particular species comprised of fused domains to correspond to a single, full-length protein of another species. For instance, the *E. coli* acetate CoA transferase α and β subunits has regions of homology to the N- and C-terminus of human succinyl CoA transferase [60], [61]. One of the most fascinating questions that can be asked of these biologically related proteins is whether they originated from a primordial fused protein separated through evolution or *vice versa.* An example of the latter, evolved by gene fusion, is the *Arom* locus of *A. nidulans* that encodes a pentafunctional polypepetide. The AROM complex consists of domains analogous to five bacterial enzymes (AroA, AroB, AroE, AroK and AroL) responsible for catalyzing polyaromatic amino acid biosynthesis [61], [62]. This AROM example affirms the fact that fused domains can retain its specific enzymatic activity; indeed, multiple lines of evidence have demonstrated that fusion events may even increase the functional efficiency of the fusion protein [61], [63].

Recent metabolic engineering efforts have enabled synthetic protein scaffolds to be built that spatially recruit successive pathway enzymes in a designable manner, ultimately improving the production of the end product while lowering the overall metabolic load on the chassis organism [64]. The principle behind such metabolic pipelines is to assemble enzyme complexes that bring the active sites close together to prevent loss of intermediates or competition from other pathways. Therefore, while we were unable to adequately test the enzymatic function of *C. neoformans* allantoicase-ureidoglycolate hydrolase, we propose that this fusion may enable faster and more efficient degradation of allantoate to urea and glyoxylate, via substrate channeling.

A closer inspection of the literature revealed that the phenomenon of fusion uric acid catabolic protein is also observed in plants and legumes, where a fusion OHCU decarboxylase-HIU hydrolase is present [9], [65]. This fusion OHCU decarboxylase-HIU hydrolase protein contains enzymes that act one after another, just like allantoicase-ureidoglycolate hydrolase. An intriguing consideration to come out of these observations is that these fusions may suggest protein-protein interactions between the multiple enzymes of this pathway. In support of this notion, allantoinase and allantoicase have been shown to form a complex in amphibians [10]. Further studies into the enzyme structures and protein-protein interactions of members of the uric acid pathway may provide insights into how *C. neoformans* has undergone evolutionary biochemical adaptation for optimized utilization of this abundant nitrogen source found in pigeon excreta.

In fact, uric acid is also a common metabolite found at concentrations as high as 2,696 μM in the serum of healthy individuals [66]. This occurrence is caused by the inactivation of the urate oxidase gene of hominoids some 15 million years ago in a primate ancestor [1]. The subsequent inactivation of other catabolic genes of the pathway is a possible evolutionary scenario; the human HIU hydrolase gene has several inactivating mutations while the OHCU decarboxylase gene, although potentially encoding a complete protein, does not appear to be transcriptionally expressed [9]. Recently, necrotic mammalian cells have been shown to release uric acid as a danger signal to enhance immune response against invading microbes [30]. Such immunomodulating properties of uric acid are not likely to be exhibited in the immunocompetent BALB/c mice, commonly used as an animal model system for testing cryptococcosis, since their biological uric acid catabolic enzymes are intact. Hence, in order to better mimic the infection of humans, we are currently in the process of breeding the C57BL/6J urate oxidase-deficient (hyperuricemia) mice colonies previously created by Wu *et al.,* to further investigate the virulence capacities of the *C. neoformans* uric acid catabolic mutants [67]. In support of our proposition, studies have shown that AIDS patients, particularly those undergoing antiretroviral therapies, have a higher prevalence of hyperuricaemia [68]. Overall, the results we report here represent the first definitive characterization of the complete uric acid degradation pathway in a second phylum of the kingdom fungi, the *Basidiomycota,* and we have laid the foundation for future research into investigating *C. neoformans* virulence via purine metabolism.

## Supporting Information

Figure S1 **ClustalW sequence alignment of** ***A. nidulans*** **UaZ and** ***C. neoformans*** **Uro1.** Identical amino acid residues are shaded dark grey while similar residues are shaded light grey. The conserved N-terminus sequence needed for enzymatic activity is boxed in red.
(DOC)

Figure S2 **ClustalW sequence alignment of** ***A. nidulans*** **UaX and** ***C. neoformans*** **Uro2.** Identical amino acid residues are shaded dark grey while similar residues are shaded light grey. The conserved C-terminus YRGS motif that distinguishes members of the transthyretin family is boxed in red. The residues indicated with an asterisk below the sequence are conserved active site residues, as determined in the structures of the enzymes from *B. subtilus, Salmonella dublin* and *Danio rerio.*
(DOC)

Figure S3 **ClustalW sequence alignment of** ***A. nidulans*** **UaW and** ***C. neoformans*** **Uro3.** Identical amino acid residues are shaded dark grey while similar residues are shaded light grey.
(DOC)

Figure S4 **ClustalW sequence alignment of** ***S. cerevisiae*** **Dal1 and** ***C. neoformans*** **Dal1.** Identical amino acid residues are shaded dark grey while similar residues are shaded light grey.
(DOC)

Figure S5 **The** ***C. neoformans DAL2,3,3*** **gene encodes a fusion allantoicase-ureidoglycolate hydrolase protein. A.** Representative protein architecture of *S. cerevisiae* Dal2 and Dal3, and *C. neoformans* Dal2,3,3. **B.** ClustalW sequence alignment of *S. cerevisiae* Dal2 and *C. neoformans* Dal2. **C.** ClustalW multiple sequence alignment of *S. cerevisiae* Dal3 and *C. neoformans* Dal3a and Dal3b. Identical amino acid residues are shaded dark grey while similar residues are shaded light grey.
(DOC)

Figure S6 **ClustalW sequence alignment of** ***A. nidulans*** **UreB and** ***C. neoformans*** **Ure1.** Identical amino acid residues are shaded dark grey while similar residues are shaded light grey.
(DOC)

Figure S7 **HIU hydrolase and OHCU decarboxylase activities are not entirely essential, but are required for efficient uric acid degradation in** ***C. neoformans*** **.** Tenfold spot dilution assays for nitrogen source utilization showed that the *uro1Δ, uro2Δ* and *uro3Δ* mutants exhibited obvious growth defects on YNB supplemented with 10 mM uric acid after a two-day incubation period, however, growth of the *uro2Δ* and *uro3Δ* strains catches up on further incubation.
(DOC)

Figure S8 **Restoration of the ability to utilize uric acid and its pathway intermediates upon complementation of the uric acid catabolic deletion mutants.** Tenfold spot dilution assays for nitrogen source utilization showed that the complemented *uro1Δ + URO1, uro2Δ + URO2, uro3Δ + URO3, dal1Δ + DAL1, dal2,3,3Δ + DAL2,3,3* and *ure1Δ + URE1* strains exhibited wild-type growth on YNB supplemented with uric acid, allantoin, urea or ammonium (10 mM each).
(DOC)

Table S1 **Fungal strains used in this study.**
(DOC)

Table S2 **Primers used in this study.**
(DOC)

Table S3 **Plasmids used in this study.**
(DOC)

Table S4 ***C. neoformans*** **uric acid catabolism defective mutants identified during the** ***Agrobacterium-*** **mediated insertional mutagenic screen.**
(DOC)
