# Supplementary material for: Characterization of the Complete Uric Acid Degradation Pathway in the Fungal Pathogen Cryptococcus neoformans
Source: PLoS One. 2013 May 7;8(5):e64292. doi: 10.1371/journal.pone.0064292 (PMC3646786; doi:10.1371/journal.pone.0064292)
Supplement: Figure S1 — ClustalW sequence alignment of A. nidulans UaZ and C. neoformans Uro1. Identical amino acid residues are shaded dark grey while similar residues are shaded light grey. The conserved N-terminus sequence needed for enzymatic activity is boxed in red. (DOC) [file pone.0064292.s001.doc]

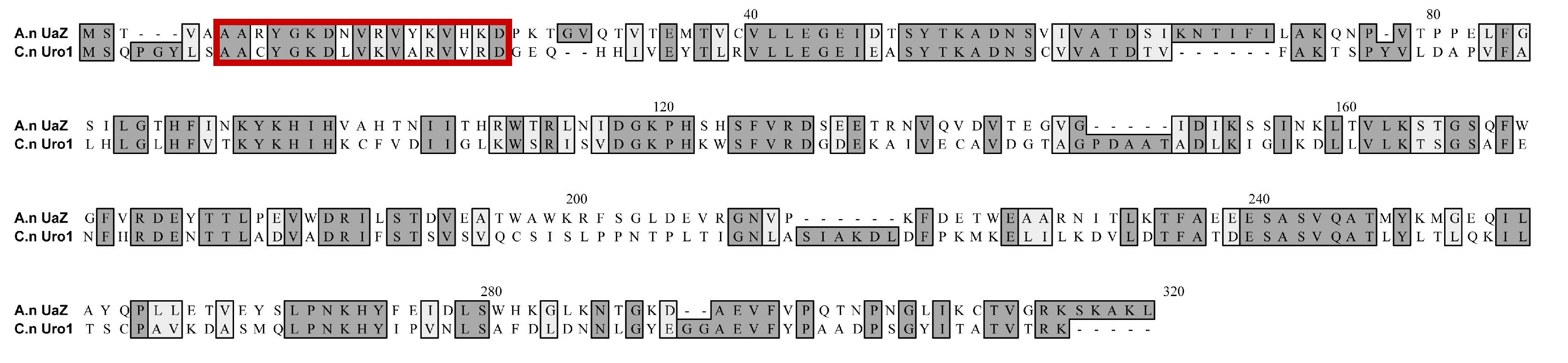


**Figure S1. ClustalW sequence alignment of *A. nidulans* UaZ and *C. neoformans* Uro1.** Identical amino acid residues are shaded dark grey while similar residues are shaded light grey. The conserved N-terminus sequence needed for enzymatic activity is boxed in red.
