# Supplementary material for: Characterization of the Complete Uric Acid Degradation Pathway in the Fungal Pathogen Cryptococcus neoformans
Source: PLoS One. 2013 May 7;8(5):e64292. doi: 10.1371/journal.pone.0064292 (PMC3646786; doi:10.1371/journal.pone.0064292)
Supplement: Figure S2 — ClustalW sequence alignment of A. nidulans UaX and C. neoformans Uro2. Identical amino acid residues are shaded dark grey while similar residues are shaded light grey. The conserved C-terminus YRGS motif that distinguishes members of the transthyretin family is boxed in red. The residues indicated with an asterisk below the sequence are conserved active site residues, as determined in the structures of the enzymes from B. subtilus, Salmonella dublin and Danio rerio. (DOC) [file pone.0064292.s002.doc]

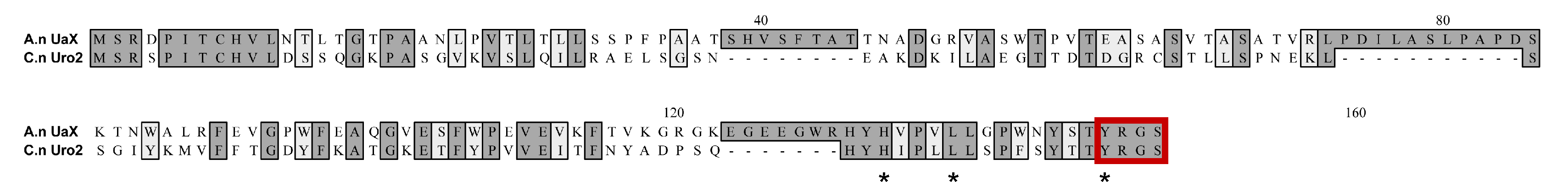


**Figure S2. ClustalW sequence alignment of *A. nidulans* UaX and *C. neoformans* Uro2.** Identical amino acid residues are shaded dark grey while similar residues are shaded light grey. The conserved C-terminus YRGS motif that distinguishes members of the transthyretin family is boxed in red. The residues indicated with an asterisk below the sequence are conserved active site residues, as determined in the structures of the enzymes from *B. subtilus, Salmonella dublin* and *Danio rerio.*
