# Supplementary material for: Characterization of the Complete Uric Acid Degradation Pathway in the Fungal Pathogen Cryptococcus neoformans
Source: PLoS One. 2013 May 7;8(5):e64292. doi: 10.1371/journal.pone.0064292 (PMC3646786; doi:10.1371/journal.pone.0064292)
Supplement: Figure S5 — The C. neoformans DAL2,3,3 gene encodes a fusion allantoicase-ureidoglycolate hydrolase protein. A. Representative protein architecture of S. cerevisiae Dal2 and Dal3, and C. neoformans Dal2,3,3. B. ClustalW sequence alignment of S. cerevisiae Dal2 and C. neoformans Dal2. C. ClustalW multiple sequence alignment of S. cerevisiae Dal3 and C. neoformans Dal3a and Dal3b. Identical amino acid residues are shaded dark grey while similar residues are shaded light grey. (DOC) [file pone.0064292.s005.doc]

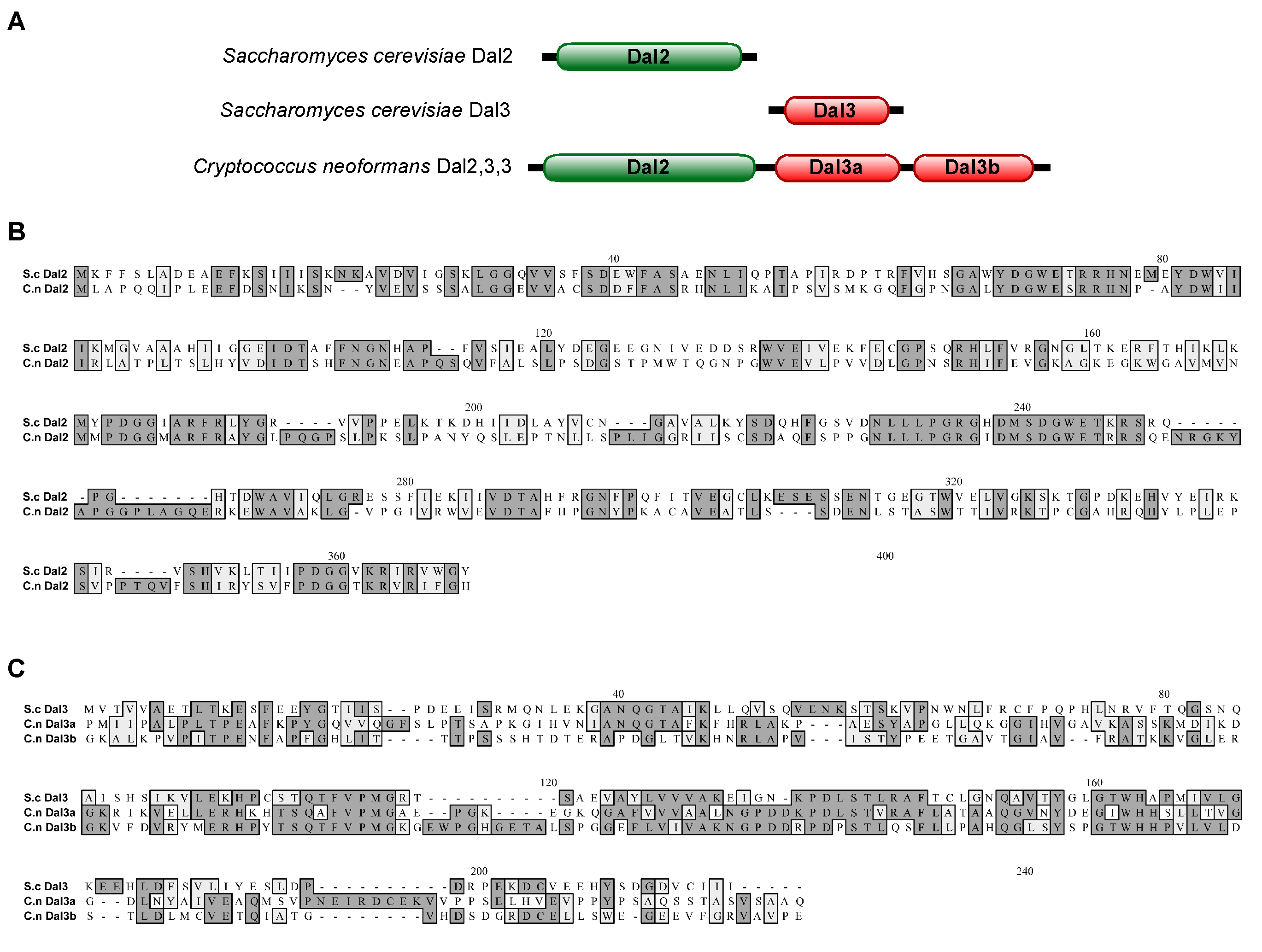


**Figure S5. The *C. neoformans DAL2,3,3* gene encodes a fusion allantoicase-ureidoglycolate hydrolase protein. A.** Representative protein architecture of *S. cerevisiae* Dal2 and Dal3, and *C. neoformans* Dal2,3,3. **B.** ClustalW sequence alignment of *S. cerevisiae* Dal2 and *C. neoformans* Dal2. **C.** ClustalW multiple sequence alignment of *S. cerevisiae* Dal3 and *C. neoformans* Dal3a and Dal3b. Identical amino acid residues are shaded dark grey while similar residues are shaded light grey.
