# Supplementary material for: Characterization of the Complete Uric Acid Degradation Pathway in the Fungal Pathogen Cryptococcus neoformans
Source: PLoS One. 2013 May 7;8(5):e64292. doi: 10.1371/journal.pone.0064292 (PMC3646786; doi:10.1371/journal.pone.0064292)
Supplement: Figure S6 — ClustalW sequence alignment of A. nidulans UreB and C. neoformans Ure1. Identical amino acid residues are shaded dark grey while similar residues are shaded light grey. (DOC) [file pone.0064292.s006.doc]

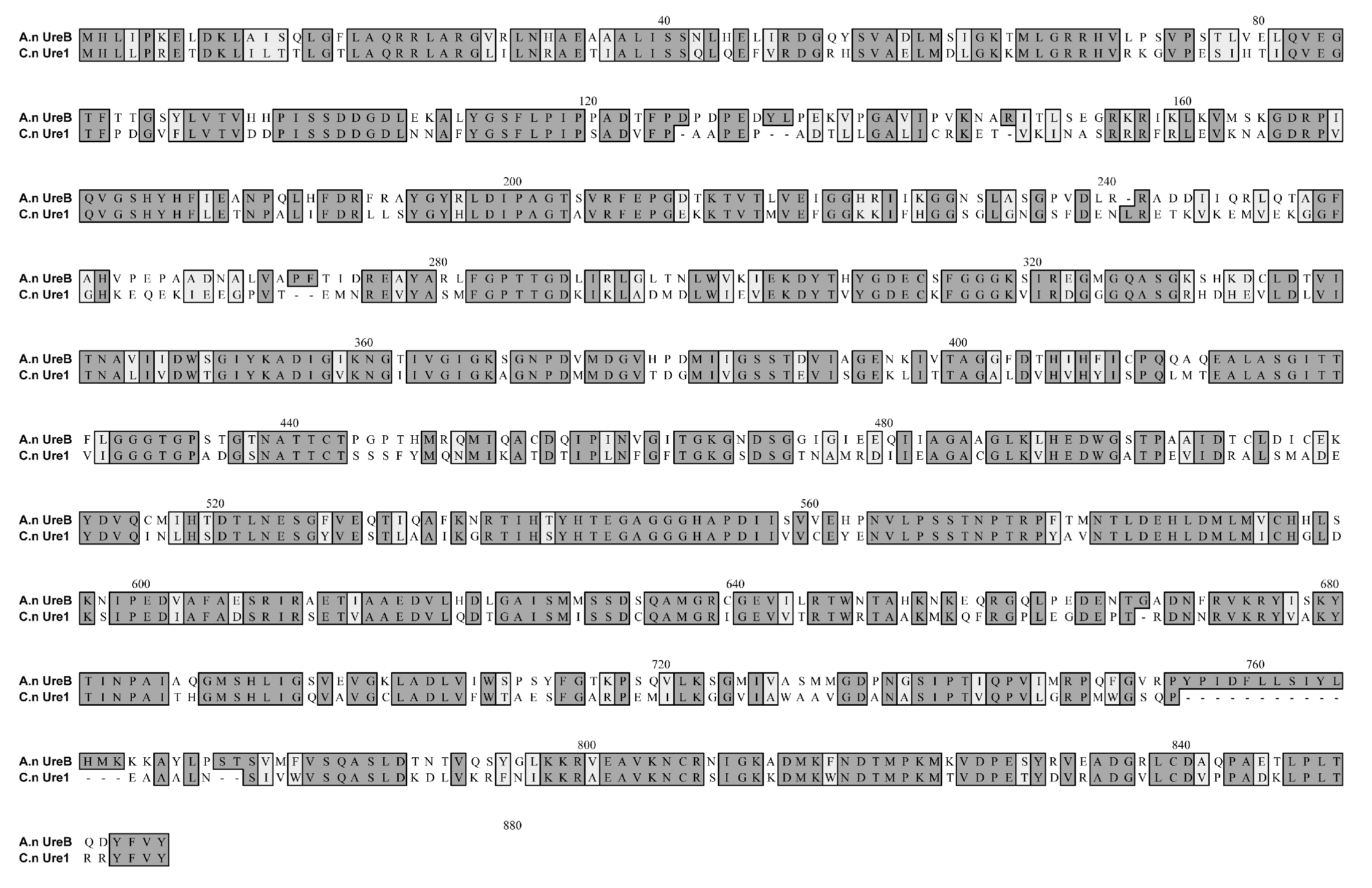


**Figure S6. ClustalW sequence alignment of *A. nidulans* UreB and *C. neoformans* Ure1.** Identical amino acid residues are shaded dark grey while similar residues are shaded light grey.
