# Supplementary material for: Characterization of the Complete Uric Acid Degradation Pathway in the Fungal Pathogen Cryptococcus neoformans
Source: PLoS One. 2013 May 7;8(5):e64292. doi: 10.1371/journal.pone.0064292 (PMC3646786; doi:10.1371/journal.pone.0064292)
Supplement: Figure S7 — HIU hydrolase and OHCU decarboxylase activities are not entirely essential, but are required for efficient uric acid degradation in C. neoformans . Tenfold spot dilution assays for nitrogen source utilization showed that the uro1Δ, uro2Δ and uro3Δ mutants exhibited obvious growth defects on YNB supplemented with 10 mM uric acid after a two-day incubation period, however, growth of the uro2Δ and uro3Δ strains catches up on further incubation. (DOC) [file pone.0064292.s007.doc]

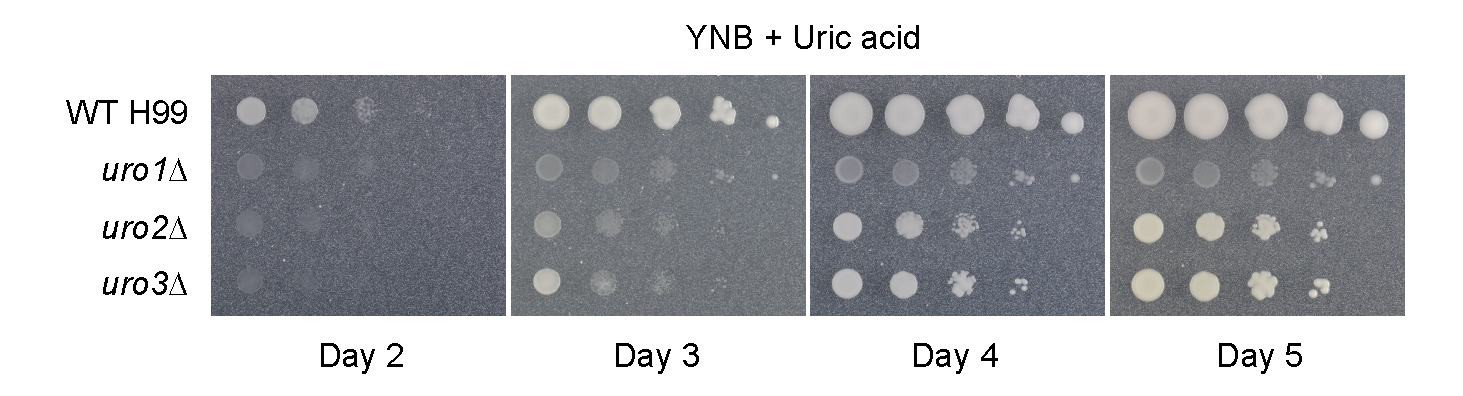


**Figure S7. HIU hydrolase and OHCU decarboxylase activities are not entirely essential, but are required for efficient uric acid degradation in *C. neoformans.*** Tenfold spot dilution assays for nitrogen source utilization showed that the *uro1Δ, uro2Δ* and *uro3Δ* mutants exhibited obvious growth defects on YNB supplemented with 10 mM uric acid after a two-day incubation period, however, growth of the *uro2Δ* and *uro3Δ* strains catches up on further incubation.
