# Supplementary material for: Characterization of the Complete Uric Acid Degradation Pathway in the Fungal Pathogen Cryptococcus neoformans
Source: PLoS One. 2013 May 7;8(5):e64292. doi: 10.1371/journal.pone.0064292 (PMC3646786; doi:10.1371/journal.pone.0064292)
Supplement: Figure S8 — Restoration of the ability to utilize uric acid and its pathway intermediates upon complementation of the uric acid catabolic deletion mutants. Tenfold spot dilution assays for nitrogen source utilization showed that the complemented uro1Δ + URO1, uro2Δ + URO2, uro3Δ + URO3, dal1Δ + DAL1, dal2,3,3Δ + DAL2,3,3 and ure1Δ + URE1 strains exhibited wild-type growth on YNB supplemented with uric acid, allantoin, urea or ammonium (10 mM each). (DOC) [file pone.0064292.s008.doc]

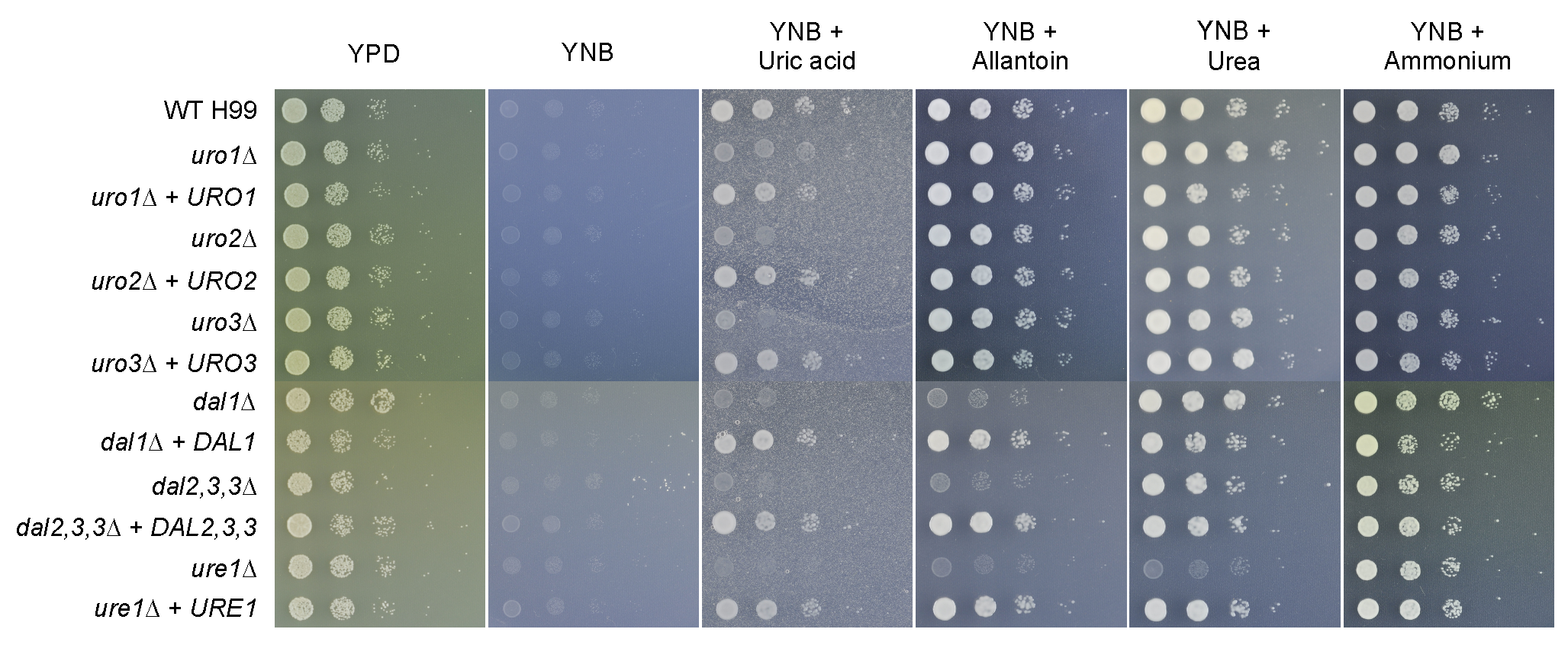


**Figure S8. Restoration of the ability to utilize uric acid and its pathway intermediates upon complementation of the uric acid catabolic deletion mutants.** Tenfold spot dilution assays for nitrogen source utilization showed that the complemented *uro1Δ + URO1, uro2Δ + URO2,* *uro3Δ + URO3, dal1Δ + DAL1, dal2,3,3Δ + DAL2,3,3* and *ure1Δ + URE1* strains exhibited wild-type growth on YNB supplemented with uric acid, allantoin, urea or ammonium (10 mM each).
