# Supplementary material for: Characterization of the Complete Uric Acid Degradation Pathway in the Fungal Pathogen Cryptococcus neoformans
Source: PLoS One. 2013 May 7;8(5):e64292. doi: 10.1371/journal.pone.0064292 (PMC3646786; doi:10.1371/journal.pone.0064292)
Supplement: Table S1 — Fungal strains used in this study. (DOC) [file pone.0064292.s009.doc]

**Table S1. Fungal strains used in this study.**

| **Strain** | **Genotype** | **Source** |
| --- | --- | --- |
| H99 | *Cryptococcus neoformans* laboratory strain | [1] |
| YL1 | H99 *uro1::NEO* | This study |
| GS1 | H99 *uro2::NEO* | This study |
| GS5 | H99 *uro3::NEO* | This study |
| EL24 | H99 *dal1::NEO* | This study |
| YL4 | H99 *dal2,3,3::NEO* | This study |
| YL5 | H99 *ure1::NEO* | This study |
| RL22 | H99 *uro1::NEO + URO1 NAT* | This study |
| RL23 | H99 *uro2::NEO + URO2 NAT* | This study |
| RL15 | H99 *uro3::NEO + URO3 NAT* | This study |
| RL13 | H99 *dal1::NEO + DAL1 NAT* | This study |
| RL16 | H99 *dal2,3,3::NEO + DAL2,3,3 NAT* | This study |
| RL17 | H99 *ure1::NEO + URE1 NAT* | This study |

**Reference**

1. Toffaletti DL, Rude TH, Johnston SA, Durack DT, Perfect JR (1993) Gene transfer in *Cryptococcus neoformans* by use of biolistic delivery of DNA. J Bacteriol 175: 1405-1411.
