# Supplementary material for: Characterization of the Complete Uric Acid Degradation Pathway in the Fungal Pathogen Cryptococcus neoformans
Source: PLoS One. 2013 May 7;8(5):e64292. doi: 10.1371/journal.pone.0064292 (PMC3646786; doi:10.1371/journal.pone.0064292)
Supplement: Table S2 — Primers used in this study. (DOC) [file pone.0064292.s010.doc]

**Table S2. Primers used in this study.**

| **Primer** | **Purpose** | **Sequence (5’-3’)** |
| --- | --- | --- |
| UQ270 | GenomeWalker adaptor | GTAATACGACTCACTATAGGGC |
| UQ271 | GenomeWalker nested adaptor | ACTATAGGGCACGCGTGGT |
| UQ272 | GenomeWalker 3’ gene specific | CTGAATGGCGAATGAGCTTGAGCTTGG |
| UQ273 | GenomeWalker 3’ nested gene specific | ATCAGATTGTCGTTTCCCGCCTTCAG |
| UQ274 | GenomeWalker 5’ gene specific | GAGAGGCGGTTTGCGTATTGGCTAGAG |
| UQ275 | GenomeWalker 5’ nested gene specific | ACGTCCGCAATGTGTTATTAAGTTGTC |
| UQ248 | *URO1* deletion 5’ F | ACATTGTTGGTAAAGCCTTCG |
| UQ249 | *URO1* deletion 5’ R | AGCTCACATCCTCGCAGCTATTGCTGATGGGTGTTGTTG |
| UQ250 | *URO1* deletion 3’ F | CCGTGTTAATACAGATAAACCTATAGATATGCAGGTGCAAAG |
| UQ307 | *URO1* deletion 3’ R | AGTAACAACCGTGGCTGTCAG |
| UQ276 | *URO1* deletion *NEO* F | ACAACACCCATCAGCAATAGCTGCGAGGATGTGAGCTGGAGAGCG |
| UQ277 | *URO1* deletion *NEO* R | CTGCATATCTATAGGTTTATCTGTATTAACACGGAAGAGATGTAG |
| UQ670 | *URO2* deletion 5’ F | TTCTGGAGTTTTCGATCCACG |
| UQ671 | *URO2* deletion 5’ R | AGCTCACATCCTCGCAGCACTGGAGACGCGATTGAT |
| UQ672 | *URO2* deletion 3’ F | TGTTAATACAGATAAACCGATCGATACTGTGTTATA |
| UQ673 | *URO2* deletion 3’ R | CCTTTGATGATACCTTTGTGA |
| UQ750 | *URO2* deletion *NEO* F | ATCAATCGCGTCTCCAGTGCTGCGAGGATGTGAGCTGGAGAGCG |
| UQ751 | *URO2* deletion *NEO* R | TATAACACAGTATCGATCGGTTTATCTGTATTAACACGGAAGAGATGTAG |
| UQ674 | *URO3* deletion 5’ F | GCCAAACCTCAAGGTTAGTCT |
| UQ675 | *URO3* deletion 5’ R | AGCTCACATCCTCGCAGCGATGAGCGTATGCGTGAT |
| UQ676 | *URO3* deletion 3’ F | TGTTAATACAGATAAACCAGCTTTAGATAGATGCTA |
| UQ677 | *URO3* deletion 3’ R | GGTTAGAAAACTGATCTGCCA |
| UQ752 | *URO3* deletion *NEO* F | ATCACGCATACGCTCATCGCTGCGAGGATGTGAGCTGGAGAGCG |
| UQ753 | *URO3* deletion *NEO* R | TAGCATCTATCTAAAGCTGGTTTATCTGTATTAACACGGAAGAGATGTAG |
| UQ236 | *DAL1* deletion 5’ F | ACCAAGCCCAAACAGAGCAGC |
| UQ237 | *DAL1* deletion 5’ R | AGCTCACATCCTCGCAGCGAACATAACGGATCAATCTCG |
| UQ238 | *DAL1* deletion 3’ F | CCGTGTTAATACAGATAAACCGCTGCACAGGAGACATTACGG |
| UQ239 | *DAL1* deletion 3’ R | GTCGGTCTTGGCGGAGAGGTG |
| UQ754 | *DAL1* deletion *NEO* F | TTTTTCTGCATACAAACAGCTGCGAGGATGTGAGCTGGAGAGCG |
| UQ755 | *DAL1* deletion *NEO* R | TAATGTCTCCTGTGCAGCGGTTTATCTGTATTAACACGGAAGAGATGTAG |
| UQ240 | *DAL2,3,3* deletion 5’ F | GCCACTGCCCAAGCTATTGAC |
| UQ278 | *DAL2,3,3* deletion 5’ R | CGCTCTCCAGCTCACATCCTCGCAGCTGATATAAGCTGTGGGGGACG |
| UQ279 | *DAL2,3,3* deletion 3’ F | CATCTCTTCCGTGTTAATACAGATAAACCATAAACGTGTTGAGAAAGCGT |
| UQ243 | *DAL2,3,3* deletion 3’ R | CGCCTAATAACACAGCAAATA |
| UQ756 | *DAL2,3,3* deletion *NEO* F | CCCCCACAGCTTATATCAGCTGCGAGGATGTGAGCTGGAGAGCG |
| UQ757 | *DAL2,3,3* deletion *NEO* R | CTTTCTCAACACGTTTATGGTTTATCTGTATTAACACGGAAGAGATGTAG |
| UQ244 | *URE1* deletion 5’ F | ACACCAACTACCGCCATTTCC |
| UQ762 | *URE1* deletion 5’ R | AGCTCACATCCTCGCAGCGTATCCGGGCTCGTGTAT |
| UQ246 | *URE1* deletion 3’ F | CCGTGTTAATACAGATAAACCGTATCAGTGCATTTCTCCATT |
| UQ247 | *URE1* deletion 3’ R | TGTGTTCCACAGCGTTGAGAG |
| UQ758 | *URE1* deletion *NEO* F | ATACACGAGCCCGGATACGCTGCGAGGATGTGAGCTGGAGAGCG |
| UQ759 | *URE1* deletion *NEO* R | GGAGAAATGCACTGATACGGTTTATCTGTATTAACACGGAAGAGATGTAG |
| UQ1323 | *URO1* genomic DNA sequencing | TCTTCCGCTTAACGACTGGAC |
| UQ1324 | *URO1* genomic DNA sequencing | CGCACGAGTCGTCCGTGATGG |
| UQ1325 | *URO1* genomic DNA sequencing | ACGGCAAGCCTCACAAATGGT |
| UQ1326 | *URO1* genomic DNA sequencing | ATTTTGACTTCATGCCCTGCC |
| UQ1327 | *URO2* genomic DNA sequencing | TATAAAATCAAATAAAAGGTG |
| UQ1328 | *URO2* genomic DNA sequencing | CATCGCAGTGTTAGATTCGTC |
| UQ1329 | *URO2* genomic DNA sequencing | GCATTGTAATTTTAAGCAAGA |
| UQ1330 | *URO3* genomic DNA sequencing | CTGTTCTTTGCGACGCTTGTT |
| UQ1331 | *URO3* genomic DNA sequencing | CCATACTCTGCTTGTTCCATC |
| UQ1332 | *URO3* genomic DNA sequencing | CGTTTGGCTGATCGGAAGGGC |
| UQ1333 | *DAL1* genomic DNA sequencing | AGATCGTATTTCCAATCCGTC |
| UQ1334 | *DAL1* genomic DNA sequencing | GCGTTTTCGTGATTTTGGATT |
| UQ1335 | *DAL1* genomic DNA sequencing | GCCAAAGAGGTCGGGGTGAGC |
| UQ1336 | *DAL1* genomic DNA sequencing | CCTCTACTGCAATCTGCCTTT |
| UQ1337 | *DAL1* genomic DNA sequencing | CTTGTAAGTCGGGCGGTGTCT |
| UQ1338 | *DAL2,3,3* genomic DNA sequencing | GGCGTGGTTCTGCTCATTGGA |
| UQ1339 | *DAL2,3,3* genomic DNA sequencing | CGTCCCCCACAGCTTATATCA |
| UQ1340 | *DAL2,3,3* genomic DNA sequencing | CCTGGAGAGGCTTTCCTGTTA |
| UQ1341 | *DAL2,3,3* genomic DNA sequencing | TCTTCTCCCTGGTCGTGGAAT |
| UQ1342 | *DAL2,3,3* genomic DNA sequencing | TCCCGCTCTGCCTCTTACCCC |
| UQ1343 | *DAL2,3,3* genomic DNA sequencing | GCGGAGTGAGTGCGACACAAG |
| UQ1344 | *DAL2,3,3* genomic DNA sequencing | GGCACCCTTATACCAGTCAGA |
| UQ1345 | *DAL2,3,3* genomic DNA sequencing | CGAATGATTGAATACTACAGT |
| UQ1346 | *URE1* genomic DNA sequencing | ATTCATCTGCATTCTGGATTA |
| UQ1347 | *URE1* genomic DNA sequencing | ATTTTTTGCTTTCCCTTTCTT |
| UQ1348 | *URE1* genomic DNA sequencing | TTCCCTGCTGCACCCGAGCCA |
| UQ1349 | *URE1* genomic DNA sequencing | ACAACTGGCGATAAGATCAAA |
| UQ1350 | *URE1* genomic DNA sequencing | TCTCTAGTAGGATGGGGATCA |
| UQ1351 | *URE1* genomic DNA sequencing | TCGTTGTCTGTGAATATGAAA |
| UQ1352 | *URE1* genomic DNA sequencing | AGATGATCCTCAAGGGCGGTG |
| UQ1353 | *URE1* genomic DNA sequencing | AAACCTCTAGTTATCTTCCAA |
