# Supplementary material for: Characterization of the Complete Uric Acid Degradation Pathway in the Fungal Pathogen Cryptococcus neoformans
Source: PLoS One. 2013 May 7;8(5):e64292. doi: 10.1371/journal.pone.0064292 (PMC3646786; doi:10.1371/journal.pone.0064292)
Supplement: Table S3 — Plasmids used in this study. (DOC) [file pone.0064292.s011.doc]

**Table S3.** Plasmids used in this study.

| **Plasmid** | **Detail** | **Source** |
| --- | --- | --- |
| pJAF1 | *NEO* selectable marker in pCR2.1-TOPO | [1] |
| pCH233 | *NAT* selectable marker in pCR2.1-TOPO | A gift from Christina Hull |
| pIRL5 | H99 *URO1* genomic DNA including promotor and terminator in pCR2.1-TOPO | This study |
| pIRL6 | H99 *URO2* genomic DNA including promotor and terminator in pCR2.1-TOPO | This study |
| pIRL7 | H99 *URO3* genomic DNA including promotor and terminator in pCR2.1-TOPO | This study |
| pIRL8 | H99 *DAL1* genomic DNA including promotor and terminator in pCR2.1-TOPO | This study |
| pIRL9 | H99 *DAL2,3,3* genomic DNA including promotor and terminator in pCR2.1-TOPO | This study |
| pIRL10 | H99 *URE1* genomic DNA including promotor and terminator in pCR2.1-TOPO | This study |
| pIRL13 | H99 *URO1* genomic DNA including promotor and terminator in pCH233 | This study |
| pIRL14 | H99 *URO2* genomic DNA including promotor and terminator in pCH233 | This study |
| pIRL15 | H99 *URO3* genomic DNA including promotor and terminator in pCH233 | This study |
| pIRL16 | H99 *DAL1* genomic DNA including promotor and terminator in pCH233 | This study |
| pIRL17 | H99 *DAL2,3,3* genomic DNA including promotor and terminator in pCH233 | This study |
| pIRL18 | H99 *URE1* genomic DNA including promotor and terminator in pCH233 | This study |

**Reference**

1. Fraser JA, Subaran RL, Nichols CB, Heitman J (2003) Recapitulation of the sexual cycle of the primary fungal pathoge*n Cryptococcus neoforma*ns var*. gatt*ii: implications for an outbreak on Vancouver Island, Canada. Eukaryot Cell 2: 1036-1045.
