# Supplementary material for: Characterization of the Complete Uric Acid Degradation Pathway in the Fungal Pathogen Cryptococcus neoformans
Source: PLoS One. 2013 May 7;8(5):e64292. doi: 10.1371/journal.pone.0064292 (PMC3646786; doi:10.1371/journal.pone.0064292)
Supplement: Table S4 — C. neoformans uric acid catabolism defective mutants identified during the Agrobacterium- mediated insertional mutagenic screen. (DOC) [file pone.0064292.s012.doc]

**Table S4.** *C. neoformans* uric acid catabolism defective mutants identified during the *Agrobacterium-*mediated insertional mutagenic screen.

| **Transformant** | **Gene disrupted** | **Insertion site** |
| --- | --- | --- |
| 65H07 | CNAG_04307.2 *(URO1)* | Promoter |
| 3H06 | CNAG_04307.2 *(URO1)* | CDS |
| 28E09 | CNAG_04307.2 *(URO1)* | CDS |
| 7H04 | CNAG_06694.2 *(URO2)* | Promoter |
| 7H05 | CNAG_06694.2 *(URO2)* | Promoter |
| 53C07 | CNAG_01108.2 *(DAL2,3,3)* | Intron |
| 88A05 | CNAG_05540.2 *(URE1)* | Promoter |
| 88A06 | CNAG_05540.2 *(URE1)* | Promoter |
